# Are we doing good? Perceived emotion regulation success and relationship quality in couples

**DOI:** 10.3389/fpsyg.2025.1683846

**Published:** 2025-12-18

**Authors:** Yan Li, Ute Kunzmann, Philipp Kanske, Margund K. Rohr

**Affiliations:** 1 Department of Psychology, Faculty of Medicine, University of Helsinki, Helsinki, Finland; 2 Institute of Clinical Psychology and Psychotherapy, Faculty of Psychology, TUD Dresden University of Technology, Dresden, Germany; 3 Wilhelm Wundt Institute for Psychology, University of Leipzig, Leipzig, Germany; 4 Department of Psychology, Faculty of Psychology and Educational Sciences, Babeş-Bolyai University, Cluj-Napoca, Romania; 5 Professorship of Developmental Psychology and Educational Psychology, Evangelische Hochschule Dresden, Dresden, Germany

**Keywords:** couples, emotion regulation success, age differences, relationship quality, gender difference, interpersonal emotion regulation

## Abstract

**Introduction:**

Emotion regulation (ER) plays a central role in shaping relationship quality and stability. However, little is known about how individuals’ perceptions of their own ER success (as regulators), their partner’s ER success (as targets), and the similarity of these perceptions relate to relationship quality across gender and age groups.

**Methods:**

The study investigated these associations in a dyadic sample of 37 younger couples (*M*_age_ = 24.33) and 41 older couples (*M*_age_ = 70.27). Both partners reported their perceived ER success and relationship quality. Bayesian structural equation modeling (BSEM) was used to examine actor, partner, and similarity effects across gender and age.

**Results:**

Higher perceived ER success was associated with greater relationship quality. This association was particularly evident among women and older ones. Among men, similarity between their perceived success as regulators and targets emerged as a unique predictor of their higher relationship quality.

**Discussion:**

These findings advance the understanding of gender- and age-specific emotional processes in romantic relationships and suggest that fostering women’s emotional self-awareness–especially in later life–and enhancing mutual emotional understanding among men can strengthen relationship quality.

## Introduction

Emotion regulation (ER) encompasses processes through which individuals influence the experience and expression of emotions–either their own or those of others (Bloch et al., 2014; Gross, 2015). When such regulation occurs within social contexts, it extends beyond the intrapersonal level and enters the domain of interpersonal emotion regulation, which involves attempts to manage emotions through social interaction (Hofmann, 2014; Niven et al., 2009; Zaki and Craig Williams, 2013). Within romantic relationships, these processes include efforts to modulate one’s own and one’s partner’s emotions, both of which have been identified as key contributors to relationship quality and stability (Bloch et al., 2014; Mazzuca et al., 2019; Parkinson et al., 2016). A growing body of research moving away from the long-dominant individual-centered view of ER shows that partners’ successful regulation of negative and positive emotions is associated with both partners’ well-being, emotional closeness and intimacy, and relationship satisfaction (Battaglini et al., 2025; Lemay et al., 2025; Niven et al., 2024; Parkinson et al., 2016; Ruan et al., 2020, 2023). For example, relationship satisfaction increases when a partner is perceived as making greater efforts to understand and take the other’s perspective (Erle and Topolinski, 2017; Lemay et al., 2007). Similarly, interpersonal ER strategies, such as emotional expression, are associated with perceived responsiveness and interaction quality (Ruan et al., 2020; Tran et al., 2024). Interestingly, little is known about perceptions of ER success from the regulator and target perspectives and how these perceptions relate to relational outcomes such as relationship satisfaction.

### Perceived ER success and relationship quality

The actor-partner interdependence model (APIM) highlights that well-being in close relationships depends on one’s own behaviors and perceptions (actor effects) and those of one’s partner (partner effects) (Cook and Kenny, 2005; Kenny and Cook, 1999; Schoebi, 2008; Sels et al., 2016; Wickham and Knee, 2012). Building on this view, interpersonal ER is best understood as a dyadic process rather than an individual one, emphasizing the inherently reciprocal nature of partners’ attempts to manage each other’s emotions (Butler and Randall, 2013; Indumathy and Kanth, 2025). ER between partners is understood as involving dynamic, reciprocal adjustments through which partners sustain emotional balance and intimacy (Butler and Randall, 2013; Zhu et al., 2025). From this perspective, individuals in interpersonal ER contexts–including, but not limited to, romantic couples–can alternate between two complementary roles: the regulator, who attempts to influence another person’s emotions, and the target, who experiences these regulation attempts.

From the regulator’s perspective, effectively managing one’s own emotions and influencing a partner’s emotions are critical for maintaining relationship quality (Ruan et al., 2023). Successful downregulation of negative emotions during disagreements or disappointments prevents escalation of conflict and preserves closeness (e.g., Bloch et al., 2014; Coan, 2008; Kappas, 2011; Tani et al., 2015). This aligns with research on extrinsic ER (i.e., regulation of another person’s emotions), and positive extrinsic ER is associated with higher dyadic adjustment and marital quality (Debrot et al., 2013; Kinkead et al., 2022, 2023; Nozaki and Mikolajczak, 2020). In addition, individuals who feel more confident in their ability to resolve conflicts tend to interpret their partner’s behaviors in a more favorable way and report greater marital satisfaction (Cui et al., 2008; Johnson and Anderson, 2013).

From the target’s perspective, the recipient’s experience of being successfully regulated by one’s partner is crucial for relationship outcomes. Related evidence suggests that such perceptions matter: for instance, perceived empathic effort from the partner predicts relationship satisfaction more strongly than actual empathic accuracy (Cohen et al., 2012), and perceiving that one’s partner takes their perspective fosters feelings of being understood and supported (Erle and Topolinski, 2017). Similarly, co-experiencing and amplifying positive emotions has been linked to greater couple cohesion and togetherness (Gable et al., 2006). While these processes are not identical to perceptions of regulation success, they suggest that subjective appraisals of partners’ emotional effort markedly influence relationship quality. However, direct evidence connecting perceived regulation success, in terms of the regulator and target perspectives, to relationship outcomes remains relatively limited and underexplored.

Recent studies have demonstrated associations between interpersonal ER success and relationship outcomes (Battaglini et al., 2025; Lemay et al., 2025; Zhu et al., 2025). Moreover, a recent daily diary study by Ruan et al. (2023) suggests that perspective is important. In this study, the perceived effectiveness of the regulators was associated with their relationship satisfaction, but not with the targets’ satisfaction, even when the targets considered ER effective. Building on this first evidence, the present study explicitly examines how subjective perceptions of regulatory success, as experienced by regulators and targets, are associated with relationship quality. We hypothesized that higher levels of perceived success as a regulator and target will be associated with higher levels of both partners’ relationship quality.

Discussions on how ER success should be defined and measured (Niven et al., 2024) are ongoing, with some scholars emphasizing changes in emotional outcomes (Bigman et al., 2016), others focusing on goal attainment in intrapersonal or interpersonal contexts (Springstein and English, 2024), and still others highlighting subjective perceptions of effectiveness (Mohammed et al., 2021). These approaches are not always consistent with one another: for instance, a strategy may reduce negative affect in the short term but fail to serve longer-term relational goals, or regulators and targets may diverge in their evaluations of the same interaction (Walker et al., 2025). While prior work has established a general positive link between regulation success and relationship quality (Bloch et al., 2014; Rompilla et al., 2022; Tani et al., 2015), little is known about how regulator and target perceptions of regulation success jointly contribute to couples’ relational outcomes. To address these gaps, we adopt a subjective perspective, examining how individuals evaluate their own success as regulators and their partner’s success as targets.

### Similarity in perceptions of ER and its association with relationship quality

Beyond each partner’s individual perceptions of ER, the extent to which these perceptions align may play an important role in shaping relationship quality. Evidence from related domains suggests that similarity fosters positive relational processes. For instance, perceived similarity in personality or interpersonal attributes has been associated with interpreting a partner’s behaviors and intentions in a more favorable way, which in turn is linked to higher relationship quality (Gonzaga et al., 2007; Iafrate et al., 2012; Tidwell et al., 2013). Likewise, individuals who perceive stronger emotional similarity with their partners report higher levels of perceived partner responsiveness (Sels et al., 2020), greater feelings of being understood and connected (Pinel et al., 2006), and higher relationship confidence and self-esteem (Iafrate et al., 2012). Extending this reasoning, similarity in perceived interpersonal ER success may signal that partners experience the regulatory process in similar ways, which has been associated with greater relational harmony (Sels et al., 2020). In the present study, similarity is conceptualized as the degree of alignment between two perspectives within each individual: the regulator perspective (perceiving oneself as successful in influencing the partner’s emotions) and the target perspective (perceiving one’s partner as successful in influencing one’s own emotions). Consistent with theoretical arguments that greater perceived alignment across perspectives fosters validation and intimacy (Gonzaga et al., 2007; Iafrate et al., 2012; Sels et al., 2020), we expect that higher similarity in perceived regulation success as a regulator and as a target will be positively associated with both partners’ relationship quality.

### Gender and age differences in ER and perceived success

Gender and age are two dimensions known to impact relationship satisfaction and ER abilities (Barrett and Bliss-Moreau, 2009; Luong et al., 2011); thus, considering partners’ gender and age is crucial. Women are frequently perceived and act as “capable regulators” in marriages regarding ER (Ball et al., 1995). They are widely viewed as the more emotional sex with a greater tendency to experience, express, and dwell on their emotions than men (Barrett and Bliss-Moreau, 2009). Conversely, men engage in less emotion-related talk and have greater difficulty identifying their partners’ emotions (Dindia and Allen, 1992). Concurrently, women are more likely than men to perceive positive aspects of emotional interactions, a phenomenon known as positive sentiment override (Story et al., 2007). Likewise, compared with men, similarity in ER seems to be more strongly related to women’s relationship satisfaction (Velotti et al., 2016). Given women’s role as “capable regulators” during emotional interactions and their tendency to perceive positive aspects of relationships, women’s perceived ER success may be more strongly linked to both their own and their partner’s relationship satisfaction.

Concerning age, theories of emotional aging propose age-related gains in ER competencies due to motivational changes and increased experience (Luong et al., 2011). For example, as one prominent theory, socioemotional selectivity theory (SST) suggests that, as time in life becomes more limited, older adults are more likely than younger adults to focus on close rather than peripheral relationships (Lang, 2001). They try to optimize the emotional climate in close relationships, seeking positive and avoiding negative interactions (Sharifian et al., 2022). In line with this, older couples emphasize togetherness more than younger couples (Rohr et al., 2019) and perceive their spouse’s behavior more positively (Henry et al., 2007). Older adults also report having more control about their emotional experience (Gross et al., 2007). Given these age differences in ER, perceived ER success as a sign of togetherness might also be a more important contributor to relationship quality for older couples.

### The present study

Overall, previous research has established that ER success plays an important role in couples’ relational well-being (Battaglini et al., 2025; Lemay et al., 2025; Niven et al., 2024; Ruan et al., 2020, 2023). However, it remains unclear how perceptions of ER success are experienced from the regulator and target perspectives, and whether alignment between these perspectives matters for relationship quality. Moreover, little is known about whether the associations between perceived regulation success and relationship quality differ by gender and age. Addressing these gaps, the present study investigates how partners’ perceptions of regulation success, from regulator and target perspectives, and the similarity between these two perspectives within individuals, are associated with each partner’s relationship quality. Furthermore, we explore whether these associations vary across gender and age. By addressing these gaps, the present study contributes to a clearer understanding of how perceived ER success relates to relationship quality in younger and older couples. Based on prior theoretical and empirical findings, we propose the following hypotheses (Hs):

H1 (Regulator perspective): Higher levels of perceived success as a regulator are associated with higher levels of both partners’ relationship quality. Specifically, for actor effects, one’s own perceived success as a regulator is associated with one’s own relationship quality; for partner effects, one’s own perceived success as a regulator is associated with the partner’s relationship quality.

H2 (Target perspective): Higher levels of perceived success as a target are associated with higher levels of both partners’ relationship quality. *Specifically, for actor effects, one’s own perceived success as a target is associated with one’s own relationship quality; for partner effects, one’s own perceived success as a target is associated with the partner’s relationship quality.*

H3 (Similarity): Higher levels of perceived similarity between regulator and target perspectives are associated with higher levels of both partners’ relationship quality. Specifically, for actor effects, greater similarity is associated with higher levels of one’s own relationship quality; for partner effects, greater similarity is associated with higher levels of the partner’s relationship quality.

H4 (Gender differences): Associations between perceived regulation success (from the regulator perspective, target perspective, and similarity between the two perspectives) and relationship quality are expected to differ by gender. *Specifically, the positive actor and partner effects are expected to be stronger for women than for men.*

H5 (Age differences): Associations between perceived regulation success (from the regulator perspective, target perspective, and similarity between the two perspectives) and relationship quality are expected to differ by age. *Specifically, the positive actor and partner effects are expected to be stronger for older couples than for younger couples.*

## Materials and methods

### Participants

Seventy-eight heterosexual couples participated in the study, including 37 younger couples (*M*_age_ = 24.34 years, SD = 3.63) and 41 older couples (*M*_age_ = 70.27 years, SD = 4.69). Younger couples had been together on average for about 4 years (*M* = 4.08, SD = 3.01), whereas older couples had been together for approximately 38 years (*M* = 38.31, SD = 14.86). Most older couples (87.8%) were cohabitating and married, compared to 8.1% of younger couples. Furthermore, most younger couples had no children (91.9%), while the majority of older couples had at least one child (97.6%, *M* = 1.80, SD = 0.72). Younger participants also reported better subjective health (*M* = 4.24, SD = 0.72) than older participants (*M* = 3.44, SD = 0.88). Moreover, the sample is relatively well-educated, as the majority have *Abitur*, which corresponds to ISCED 3a and qualifies one for higher education. More specifically, most men (76.9%) and women (70.5%) had completed *Abitur*. Smaller proportions reported holding a university of applied sciences entrance qualification (men: 9.0%; women: 6.4%) or an intermediate secondary school certificate (men: 9.0%; women: 16.7%), while only a few participants had a lower secondary school certificate (men: 3.8%; women: 3.8%) or another type of qualification (e.g., engineering school; men: 1.3%; women: 2.6%). In terms of employment, the majority of young adults were students (women: 81.1%, men: 64.9%), while most older adults were retired and no longer working (women: 90.2%, men: 92.7%).

### Procedure

This study employed a cross-sectional, survey-based dyadic design. Although the broader project included additional experimental tasks, the present study focuses exclusively on questionnaire. Couples were recruited from a pre-registered participant pool and through online advertisement, newspaper announcements, and flyers displayed at public places. All participating couples had been in stable, exclusive romantic relationships for at least one year. The eligible participants were heterosexual couples. Younger and older couples were recruited to ensure age variation. To participate, young adults should be between 18 and 35 years old, and older adults should be at least 50 years old. Upon arrival at the laboratory at the University of Leipzig, Germany, both partners provided written informed consent and completed initial questionnaires on demographics and current well-being. The experimental session comprised several conversational tasks. First, couples engaged in a neutral conversation to acclimate to the laboratory setting (baseline phase). Next, partners were separated: one partner relived a personally meaningful negative event (comfort seeker), while the other recalled a neutral event (comforter; reliving phase). Finally, the couple reunited to engage in a comforting interaction, during which the comforter provided support to the comfort seeker (comforting phase; for further details, see Rohr et al., 2019).

At the end of the experimental session, participants completed electronical questionnaires independently that included measures of ER, perceived success in regulating their partner’s emotions (regulator perspective), perceived success of their partner in regulating their own emotions (target perspective), and relationship quality. This part of the questionnaire reflected participants’ general reports of their relationship rather than evaluations tied to the laboratory conversation. The measures relevant to the present study were completed at this stage. Participants received either €5 per hour or course credit in the form of test subject hours. The study followed the ethical guidelines of the American Psychological Association. The procedure was approved by the Ethics Committee of the German Psychological Society (UK 112012 and Rohr 042018) and followed the Declaration of Helsinki.

### Measurements

#### Relationship quality

Relationship quality was measured using a short form of the Partnership Questionnaire (Kliem et al., 2015). To assess the current relationship quality of couples, nine items (e.g., “We talk to each other for at least half an hour in the evening”) were rated on a scale ranging from 0 (never/very rarely) to 4 (very often). The internal consistency was satisfactory in the entire sample and comparable between men and women (total sample: α = 0.89; women: α = 0.87; men: α = 0.81).

#### Perceived ER success

Perceived ER success was measured using two self-developed items, one from the regulator’s perspective and one from the target’s, which were consistent with prior research on perceived regulatory effectiveness (e.g., Côté et al., 2010). These ratings reflected participants’ general perceptions of their relationship rather than evaluations tied to the laboratory conversation. Specifically, perceived success as a regulator was measured by the item “How successful are you in influencing your partner’s feelings?” (Please see Supplementary Material 1 for further information). Perceived success as a target was measured using the item “How successful is your partner in influencing your feelings?” Participants answered on a scale ranging from 1 (not at all/a little) to 5 (extremely). This method allowed for a comprehensive assessment of perceived ER success from both perspectives within the relationship.

#### Similarity in perceptions

Similarity, conceptualized as the degree of within-person alignment, was not measured directly but was computed from two perspectives within each individual: the regulator perspective (how successful individuals perceived themselves to be in influencing their partner’s emotions) and the target perspective (how successful they perceived their partner to be in influencing their own emotions). To determine similarity in perceptions, we computed the absolute difference between the regulator and target ratings (i.e., the Euclidean distance in one-dimensional space). To facilitate interpretation, the difference scores were reverse-coded, such that higher values reflected greater similarity (smaller discrepancy) and lower values reflected lesser similarity (larger discrepancy) (Sels et al., 2020). The formula used is as follows:


Similarity=-1×|Success⁢as⁢a⁢regulator-Success⁢as⁢a⁢target|

This approach allowed us to derive a similarity score for each individual, which could then be analyzed separately for women and men.

### Covariates

We included education level, marital status, and relationship duration as covariates because prior research has shown that these factors are systematically related to ER processes and relationship outcomes (Hughes et al., 2020; McCabe et al., 1996; Mihalcea et al., 2013). We recoded education status into a dichotomous variable (0 = less than university; 1 = university degree or higher) to simplify the model. Marital status was also dichotomized (0 = not married; 1 = married). We have also included participants’ subjective health as a covariate because it was identified as a relevant variable in the larger longitudinal project. The participants’ health was assessed with a single item ranging from 1 (poor) to 5 (very good).

### Statistical analysis

RStudio (Version 4.3.3) was employed as the software platform, with the primary package utilized being blavaan (Merkle and Rosseel, 2018) and rstanarm (Gabry and Goodrich, 2016). We first conducted independent sample *t*-tests to examine age group differences in the main study variables (perceived regulation success from the regulator and target perspectives, perceived similarity, and relationship quality) using couple-level composite scores that combined both partners’ responses. In addition, paired sample *t*-tests were performed to assess gender differences in these variables. Furthermore, to test our hypotheses, we estimated APIMs (Cook and Kenny, 2005) using Bayesian structural equation modeling. This approach allows for the simultaneous estimation of actor effects (the association between one’s own predictor and one’s own outcome) and partner effects (the association between one’s partner’s predictor and one’s own outcome) while appropriately accounting for the interdependence between dyad members. Separate APIMs were estimated for perceived success from the regulator and target perspectives (H1 and H2). The effects of within-person similarity between the regulator and target perspectives on relationship quality (H3) were examined using general linear models. Gender differences (H4) were assessed within the APIM framework by including gender as a distinguishing variable, and age differences (H5) were tested via Bayesian multigroup analysis (MGA; e.g., Evermann, 2010), comparing models across younger and older couples. In line with the APIM framework, dyads were treated as distinguishable by gender (male vs. female), as recommended by Kenny et al. (2006).

Given the relatively small sample size, all models were estimated in a Bayesian framework, which provides a robust approach for small-sample research by incorporating prior information and generating full posterior distributions (Gelman and Hill, 2006; van de Schoot et al., 2015). In support of our choice, Johnson and Baldwin (2020) illustrated the use of Bayesian modeling for dyadic data (e.g., APIM) in couple and family therapy research, noting that Bayesian methods are well suited for small-to-moderate samples, provide more credible inference under uncertainty, and flexibly accommodate complex dependence structures. This approach utilizes Markov chain Monte Carlo sampling to generate posterior estimates for all parameters (Merkle and Rosseel, 2018). Parameter credibility was evaluated using 95% credible intervals (CIs), and significance was inferred when these intervals did not include zero. Model fit was evaluated using the posterior predictive *p*-value (PP*p*), where values close to 0.50 indicate a good fit between the model and the observed data, whereas values below 0.05 or 0.01 suggest poor fit (van de Schoot et al., 2014). For the MGA, following Kass and Raftery (1995), we interpreted twice the natural logarithm of the Bayes factor [2 × log(BF)] values between 6 and 10 as providing strong evidence for group differences.

As sufficient data to construct informative priors was unavailable, we followed established recommendations and used default priors (McDonald and Ho, 2002). This approach helps maintain the objectivity of the analysis and facilitates comparability with future research (Keysers et al., 2020). Based on the recommendations (McDonald and Ho, 2002), three Markov chains with 1,500 iterations each were run, of which the first 500 were discarded as warm-up, yielding 3,000 posterior draws in total. Convergence was assessed using the potential scale reduction factor (*R*); all parameters had *R* ≤ 1.01, and effective sample sizes were adequate. All primary models were first estimated without covariates and were reported as the main results. To assess the robustness of these findings, we then re-estimated all models, including all covariates, as sensitivity analyses.

The current study was not pre-registered but the data and analysis code supporting the findings of this study are available on the Open Science Framework (OSF) at: https://osf.io/ptzcd/overview.

## Results

Table 1 shows the descriptive statistics of the main variables. Correlations between all study variables are shown in Table 1 and Supplementary Material 2.

**TABLE 1:** Age and gender differences in main variables.

Variables	Younger (*n* = 37)				Older (*n* = 41)				Age and gender group differences
	Women		Men		Women		Men		
	*M*	SD	*M*	SD	*M*	SD	*M*	SD	
Success as a regulator	3.19	0.70	3.32	0.94	2.98	0.99	2.85	0.69	Age: *t*(76) = 2.30*, *p* = 0.024, *d* = 0.53
									Gender: *t*(77) = 0, *p* = 1.000
Success as a target	3.46	1.02	3.38	0.98	3.29	1.05	3.17	0.80	Age: *t*(76) = 1.16, *p* = 0.249
									Gender: *t*(77) = −0.70, *p* = 0.486
Similarity in perceptions	−0.65	0.68	−0.70	0.78	−0.71	0.84	−0.46	0.64	Age: *t*(76) = −0.64, *p* = 0.522
									Gender: *t*(77) = −1.11, *p* = 0.270
Relationship quality	3.49	0.37	3.20	0.50	2.98	0.60	2.94	0.47	Age: *t*(76) = 3.92***, *p* < 0.001, *d* = 0.89
									Gender: *t*(77) = 2.84**, *p* = 0.006, *d* = 0.30

*N* = 78 couples. *p < 0.01, **p < 0.01, ***p < 0.001.

### Associations between perceived success as regulators and relationship quality

**FIGURE 1:**
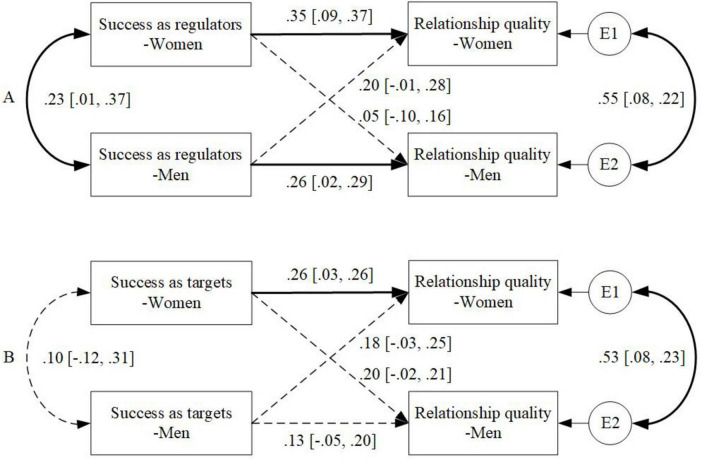
Associations of perceived emotion regulation success with relationship quality: **(A)** as regulators and **(B)** as targets. Standardized coefficients are depicted; solid lines represent statistically significant associations; dashed lines represent non-significant associations.

The Bayesian model for the associations between ER success as regulators and relationship quality is well-fitted (PP*p* = 0.499), and all parameters showed satisfactory convergence (*R* ≈ 1). As shown in Figure 1A, for the actor effects, women’s perceived success as regulators was associated with their own higher relationship quality (β = 0.35, 95% CI [0.09, 0.37]), in line with H1. Similarly, for men, the actor effect of perceived success as a regulator was associated with their own higher relationship quality (β = 0.26, 95% CI [0.02, 0.29]). However, significant partner effects did not exist for either gender. Consistent with past work on ER (Riahi et al., 2020), an additional finding was that women’s and men’s perceptions of their own success were correlated (β = 0.23, 95% CI [0.01, 0.37]), as was their reported relationship quality (β = 0.55, 95% CI [0.08, 0.22]). All effects reported in the Results are standardized estimates, which can be interpreted as correlations rather than raw covariances.

#### Associations between perceived success as targets and relationship quality

In the second step, we explored the associations between perceived success as targets and relationship quality (see Figure 1B). The Bayesian model fitted well (PP*p* = 0.511), and all parameters showed satisfactory convergence (*R* ≈ 1). Similarly, the actor effect of women’s perceived success as targets was associated with their own higher relationship quality (β = 0.26, 95% CI [0.03, 0.26]), in line with H2.

### Similarity in perceptions of regulation success and relationship quality

In line with H3, men’s within-person similarity between the regulator and target perspectives was associated with higher levels of their own relationship quality (β = 0.30, 95% CI [0.01, 0.68]), but not with their partner’s relationship quality (see Table 2). Moreover, no significant associations were found between similarity and women’s relationship quality.

**TABLE 2:** Regression analysis of similarity on relationship quality.

Variables	Predictor	β	SD	95% CI		R
				LL	UL	
Relationship quality (men)	Intercept	0.30*	0.20	0.02	0.63	1
	Similarity (men)	0.30*	0.20	0.01	0.68	1
	Similarity (women)	−0.20	0.20	−0.56	0.25	1
	Age	−0.60*	0.20	−1.01	−0.13	1
	Similarity (men) × age	−0.30	0.20	−0.81	0.19	1
	Similarity (women) × age	0.10	0.30	−0.38	0.62	1
Relationship quality (women)	Intercept	0.50*	0.20	0.22	0.82	1
	Similarity (men)	0.20	0.20	−0.07	0.56	1
	Similarity (women)	0.00	0.20	−0.40	0.36	1
	Age	−0.90*	0.20	−1.35	−0.53	1
	Similarity (men) × age	−0.20	0.20	−0.70	0.22	1
	Similarity (women) × age	0.20*	0.20	0.75	1.06	1

Number of couples = 78. CI, confidence interval; LL, lower limit; UL, upper limit.

*p < 0.01.

### Age differences in the link between perceived ER success and relationship quality

**FIGURE 2:**
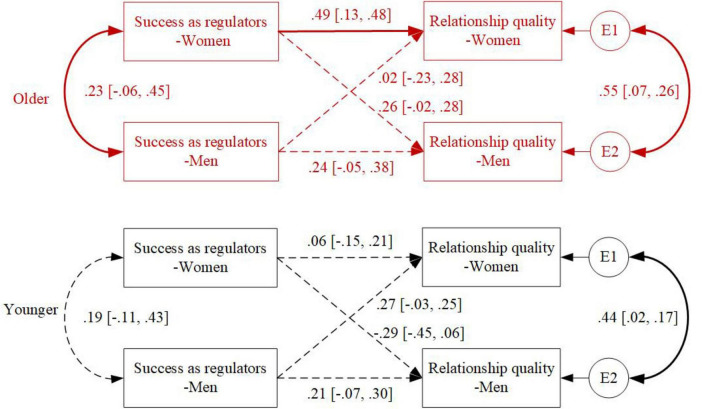
Age differences in the associations between perceived success as regulators and relationship quality. Standardized coefficients are depicted; solid lines represent statistically significant associations, dashed lines represent non-significant associations.

Regarding age differences in the associations between perceived success as regulators and relationship quality, MGA revealed that the actor effects between women’s perceived success as regulators and their own relationship quality were more significant for older couples than for younger couples (2log_e_(BF) = 8.54; Older: β = 0.49, 95% CI [0.13, 0.48]; Younger: β = 0.06, 95% CI [−0.15, 0.21]), in line with H5 (see Figure 2).

**FIGURE 3:**
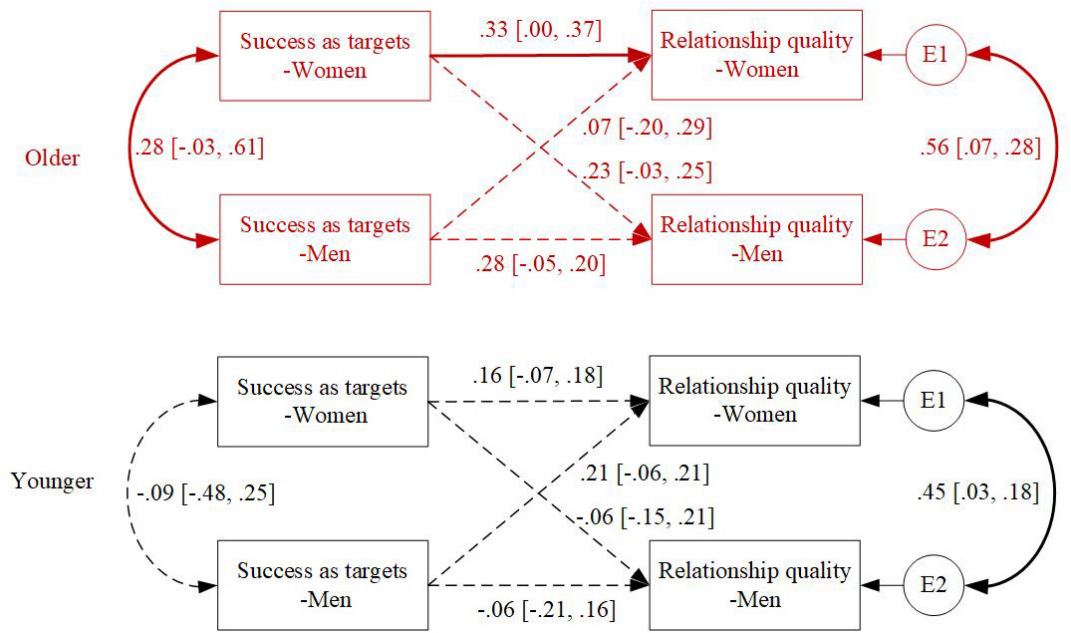
Age differences in the associations between success as targets and relationship quality. Standardized coefficients are depicted; solid lines represent statistically significant associations; dashed lines represent non-significant associations.

In examining age differences in the associations between perceived regulation success as targets and relationship quality, MGA revealed age differences in the actor effects between women’s perceived success as targets and their own relationship quality (2log_e_(BF) = 8.49) (see Figure 3 for details). Specifically, higher perceived success as targets was marginally associated with higher levels of relationship quality for older couples (β = 0.33, 95% CI [0.00, 0.37]), while it was not significant for younger couples (β = 0.16, 95% CI [−0.07, 0.18]).

Additionally, all covariates (education, marital status, relationship duration and subjective health) were included in each model to account for potential confounding factors. After controlling for these variables, the overall pattern of the findings remained robust. The only exception was the actor effect of men’s perceived success as regulators on their own relationship quality, which was reduced to non-significance (β = 0.22, 95% CI [−0.02, 0.28]) in the APIM for perceived regulator success and relationship quality (Figure 1A).

## Discussion

The present study examined the associations between individuals’ perceived emotion regulator success (as regulators and targets), the similarity of these perceptions, and both partners’ relationship quality, as well as potential gender and age differences in these associations based on Bayesian statistics. Our study yielded several noteworthy results. First, higher levels of perceived regulation success were associated with better relationship quality, particularly for women. Second, greater men’s similarity between success as regulators and targets was associated with their higher relationship quality, indicating that congruence in regulation success perceptions might be more important for males than for females. Third, the associations between perceived success and relationship quality were more pronounced in older couples than in younger ones.

### Women’s perceptions of regulation success are more closely tied to relationship quality

We found that higher levels of perceived ER success were associated with better relationship quality, particularly for women. On the one hand, women’s perceived success as regulators is linked to their own relationship quality, consistent with H1. It seems important for women to see oneself as competent and active co-producer of the relationship as this sense of accomplishment may lead to increased engagement and higher relationship quality (Schokker et al., 2010). On the other hand, in line with H2, women’s perceived success as targets is also linked to their own relationship quality, indicating that feeling their partner’s engagement during the process is crucial. This finding aligns with previous research indicating that perceived empathic effort from one’s partner is strongly related to partners’ relationship satisfaction (Cohen et al., 2012). These findings may be better understood in light of mechanisms such as emotional interdependence and fulfilled expectations. When partners perceive each other’s ER efforts as successful, they may experience greater emotional alignment and mutual responsiveness, reinforcing a shared sense of understanding and connectedness (Falconier et al., 2015; Reis et al., 2004). Moreover, perceiving one’s partner as emotionally effective may fulfill expectations of support and closeness, which are central to relationship satisfaction (Laurenceau et al., 1998). Future research could test these mechanisms directly to further elucidate how perceived ER success contributes to relationship quality. Ideally, this should be done longitudinally in order to trace bidirectional, cross-lagged correlations. Our findings add to ongoing debates on how ER success should be defined (Niven et al., 2024) by demonstrating that subjective perceptions of success–across both regulator and target perspectives–are meaningfully associated with their relationship quality. Ideally, these subjective evaluations of ER success should be recorded alongside behavioral outcomes and indicators of emotional changes (Bigman et al., 2016; Springstein and English, 2024) to investigate whether different success indicators predict different outcomes.

Interestingly, when covariates were included, the association between men’s perceived success as regulators and their own relationship quality was no longer statistically significant. One possible explanation is that structural factors such as relationship duration, marital status, and health may account for much of the variance in men’s reports of relationship quality, leaving less unique variance attributable to perceptions of regulatory success. Prior research has shown that these contextual and demographic factors are robust predictors of marital and relationship outcomes (Bookwala and Franks, 2005; Karney and Bradbury, 1995; Umberson and Karas Montez, 2010). This suggests that for men, such background factors play a stronger role in shaping relationship quality than subjective evaluations of regulation success.

In addition, consistent with H4, women’s, but not men’s, perception of regulation success was associated with their relationship quality. This aligns with findings that women tend to engage in more emotional communication and seek emotional intimacy within relationships than men (Barrett and Bliss-Moreau, 2009). This inclination may lead women to hold higher expectations for successful emotional support between partners, and they probably attribute more responsibility for shaping the relationship to themselves (Wilcox and Nock, 2006). In line with this, women and men did not differ in their evaluation of their own and their partner’s success, but women’s evaluations were more strongly tied to their relationship satisfaction. Collectively, women may prioritize their partners’ or their own ability to effectively regulate emotions, as this directly impacts the emotional climate within the relationship and subsequent assessment of relationship quality. In our study, we referred to a general perception of regulatory success. However, it would be interesting if the perceived success of ER was measured after concrete interactions in which emotions are regulated by the couples and whether these success perceptions differ in dependence on the type of situations (e.g., conflict vs. comforting situations), the emotions regulated (e.g., anger vs. sadness) and with regard to underlying goals (e.g., being assertive vs. signaling togetherness). Here, a combination of observations in the laboratory and in everyday life via ambulatory assessments would be ideal to gain a comprehensive view of the role of emotion success for the relationship and to separate partner-, relationship-specific, and situational variability.

### Similarity between the perspectives of regulators and targets enhances men’s relationship satisfaction

Consistent with H3, men who perceived greater similarity between the regulator and target perspectives reported higher relationship quality. This finding contributes to the growing literature on perceived congruence as a predictor of relationship satisfaction. Previous studies have shown that congruence in domains such as personality perceptions or interpersonal styles is linked to better relationship outcomes (Iafrate et al., 2012; Tidwell et al., 2013). Our findings extend this work by demonstrating that within-person alignment between regulator and target perspectives of ER success may serve a similar function, particularly for men.

Interestingly, in our sample and contrary to previous evidence (Velotti et al., 2016), this effect was specific to men, suggesting that while women benefit more from higher overall levels of ER success, men may be especially sensitive to how well their own regulation success perceptions align with those of their partners. This may reflect a preference for emotional coordination or shared emotional standards, which can foster a sense of mutual understanding and relational stability (Iafrate et al., 2012; Pinel et al., 2006). Thus, perception congruence in ER success appears to serve as a unique relational resource for men. Future research should further investigate the gender-specific pathways through which perceived success and its congruence influence relationship quality.

### Perceived ER success matters more in older couples

In line with H5, the association between perceived ER success and relationship quality was more pronounced in older couples than in younger couples. More concretely, older but not younger women’s success as regulators was associated with their own relationship quality. This finding is consistent with SST, which postulates a higher motivation to preserve and optimize close relationships in old age (Lang, 2001). These results underscore the increasing importance of effective ER in maintaining relationship quality as couples age. Future research could explore the specific aspects of ER that become more critical with age, such as the role of emotional support, and adaptive coping strategies, leading to more targeted interventions to support relationship satisfaction across different stages of life.

Unlike Gross et al. (2007) who found age-related increases in emotional control, older couples in our study reported lower levels of success as regulators than their younger counterparts in the mean level. One potential explanation could be cohort differences in the meaning of ER. While younger adults express their emotions more strongly and focus more on ER and relationship work, older cohorts have been socialized even more strongly not to show their emotions (Hülür and Castano, 2019). It could also be that older couples tend to become more humble (Ogihara and Kusumi, 2020), maybe downplaying their role in regulating one’s emotion.

## Limitations and directions for future research

The current study revealed the associations between perceived ER success and romantic relationship quality while exploring age and gender differences in these associations. Nevertheless, some limitations exist. First, owing to challenges in recruiting elderly couples, our sample size was relatively small, which raises concerns about the statistical power in dyadic APIM models. Although we employed Bayesian statistics to enhance the robustness of our analyses, the limited sample size may restrict the generalizability of our findings. Future studies should replicate the current results with larger samples to ensure sufficient power and more reliable estimates. Second, we assessed ER success using the wording “influence” rather than “regulate.” Although our results indicate that the item was understood in terms of ER (i.e., balancing the emotional experience in the partnership), alternatively, underlying power differences in couples could also be the reason for the associations found. More concretely, studies on relationship power during conflicts found that partners who experience themselves as powerful were more likely to experience positive emotions and relational rewards (i.e., that their partner liked them) than powerless partners (Anderson and Berdahl, 2002). One avenue for future research is to take a closer look at this association between power dynamics, emotional experience and the perception of regulatory success, for example, by explicitly measuring control and power or varying these aspects experimentally. For example, studies could compare conflict discussions with comforting conversations, as the former should trigger the negotiation of power more than the social sharing of sad events (e.g., Ben-Naim et al., 2013; Rohr et al., 2019). Third, we relied on one-time and single-item self and partner reports. It is possible, for instance, that such reports reflect the strongest feelings individuals have on a particular day, or how they feel at the time they provide the ratings (Röcke et al., 2011). Future research could include random multiple momentary assessments throughout the day or on different days to draw a more concrete picture of ER perceptions that are more context dependent (Kuppens and Verduyn, 2015). Fourth, we could not separate the effects of age and cohort in our study. More longitudinal work could be conducted to disentangle the effects of age and cohort. Fifth, the reliance on subjective reports to measure ER success introduces biases and limitations. Participants’ subjective perception may not align with objective criteria or behavioral measures, potentially impacting validity. Comparing subjective reports to objective criteria can reveal alignment or discrepancy, offering a nuanced understanding. Future research could integrate subjective and objective measures for a more comprehensive evaluation. Finally, although our measures captured general perceptions of regulatory success without distinguishing between upregulation and downregulation, prior work suggests that these processes play different roles in relationship functioning (Haase, 2023). Future research should therefore employ more nuanced measures to capture whether success refers to enhancing positive emotions, reducing negative emotions, or avoiding regulation that worsens the partner’s state.

## Conclusion

This study advances the understanding of the associations between perceived ER success, similarity in perceived success, and relationship quality in romantic couples, with a particular emphasis on age and gender differences. The findings demonstrated that perceived ER success and similarity of these regulation success are associated with relationship quality. Notably, women’s, but not men’s, perceived success as regulators was associated with their relationship quality. This suggests that intervention programs aiming to improve relationship functioning may be more effective for women when they focus on strengthening individual ER skills and enhancing self-awareness of emotional experiences. In contrast, men’s relationship quality was more closely linked to the similarity between their success as regulators and targets. Accordingly, interventions targeting men may benefit from promoting mutual emotional awareness, encouraging shared emotional goals, and fostering dyadic coordination in ER. In addition, the associations between success as regulators and relationship quality were more pronounced in older couples than in younger couples. This highlights the need for age-specific approaches in intervention programs. For older couples, interventions that emphasize ER success and mutual emotional support may be particularly beneficial in maintaining or enhancing relationship quality. This study highlights the need to further explore the potential role of perceptions in ER success in different emotional contexts, and to integrate subjective and objective views of regulation success in younger and older couples.

## Data Availability

The datasets presented in this study can be found in online repositories. The names of the repository/repositories and accession number(s) can be found below: the data and analysis code supporting the findings of this study are available on the Open Science Framework (OSF) at: https://osf.io/ptzcd/overview.
