# Supplementary material for: Are we doing good? Perceived emotion regulation success and relationship quality in couples
Source: Front Psychol. 2025 Dec 18;16:1683846. doi: 10.3389/fpsyg.2025.1683846 (PMC12756379; doi:10.3389/fpsyg.2025.1683846)
Supplement: Supplementary file 1 [file Supplementary_file_1.docx]

## Supplementary Materials

## Supplementary 1

## Further Information About Measuring Emotion Regulation Success

We decided to assess emotion regulation success using the word “influence” instead of the more academic word “regulate”. We believe that the slightly different wording is less problematic due to the embeddedness of the accordant questions in a study on conversations about emotional events. In this vein, emotion regulation success was associated with both partner’s motive of maintaining emotional balance in the relationship as assessed by a single, 5-point scaled item (own success: *r*_women_ = 0.28, p = .015; *r*_men_ = 0.27, p = .015; partner’s success: *r*_women_ = 0.40, p < .001; *r*_men_ = 0.12, p = .302). However, one researcher pointed out that it might tend to reflect power structures in the couple. Therefore, we tested this assumption by looking at how reciprocally the couples perceive their relationship. In doing so, we used the Graphic Balance Scale by Neyer and colleagues (2011) and found no association between reciprocity as a potential indicator of power dynamics and the regulation success items (own success: *r*_women_ = 0.10, p = .389; *r*_men_ = -0.08, p = .472; partner’s success: *r*_women_ = 0.04, p = .719; *r*_men_ = 0.05, p = .695).

**Supplementary 2**

**Table 1**

*Correlations Between Study variables*

| Variables | 1 | 2 | 3 | 4 | 5 | 6 | 7 | 8 | 9 | 10 | 11 | 12 |
| --- | --- | --- | --- | --- | --- | --- | --- | --- | --- | --- | --- | --- |
| 1. Regulation success as a regulator (men) | - |  |  |  |  |  |  |  |  |  |  |  |
| 2. Regulation success as a target (men) | 0.47*** | - |  |  |  |  |  |  |  |  |  |  |
| 3. Regulation success as a regulator (women) | 0.24* | 0.19. | _- |  |  |  |  |  |  |  |  |  |
| 4. Regulation success as a target (women) | 0.31** | 0.1 | 0.48*** | - |  |  |  |  |  |  |  |  |
| 5. Relationship quality (men) | 0.27* | 0.15 | 0.12 | 0.21. |  |  |  |  |  |  |  |  |
| 6. Relationship quality (women) | 0.29* | 0.2. | 0.4*** | 0.28* | 0.58*** | - |  |  |  |  |  |  |
| 7. Subjective health (men) | 0.19. | 0.03 | 0.03 | -0.08 | 0.17 | 0.14 | - |  |  |  |  |  |
| 8. Subjective health (women) | 0.14 | 0.03 | 0.04 | 0.05 | 0.21. | 0.39*** | 0.21. | - |  |  |  |  |
| 9. Age group | -0.28* | -0.12 | -0.12 | -0.08 | -0.25* | -0.46*** | -0.3** | -0.59*** | - |  |  |  |
| 10. Education level (men) | 0.09 | 0.26* | 0.22. | 0.18 | -0.01 | 0.03 | -0.04 | 0.09 | -0.16 | - |  |  |
| 11. Education level (women) | -0.02 | 0.06 | 0.12 | 0.11 | 0.22. | 0.34** | 0.15 | 0.44*** | -0.46*** | 0.18 | - |  |
| 12. Marriage status | -0.32** | -0.28* | -0.03 | -0.1 | -0.23* | -0.37*** | -0.2. | -0.51*** | 0.81*** | -0.21. | -0.26* | - |
| 13. Relationship duration | -0.25* | -0.11 | -0.06 | -0.06 | -0.26* | -0.43*** | -0.23* | -0.55*** | 0.84*** | -0.16 | -0.29* | 0.78*** |

*Note*. **p* < .05, ** *p* < .01, *** *p* < .001. For the purposes of analysis, age, education level, marital status, and relationship duration were dichotomised.
